# Graphitic Carbon Nitride: A Reusable and Stable Support for Glucose Oxidase

**DOI:** 10.1021/acs.langmuir.5c00737

**Published:** 2025-06-17

**Authors:** Rita A. M. Barros, Loan Rivereau, Maria J. Sampaio, Raquel O. Cristóvão, Cláudia G. Silva, Joaquim L. Faria

**Affiliations:** 1 LSRE-LCM − Laboratory of Separation and Reaction Engineering − Laboratory of Catalysis and Materials, Faculty of Engineering, 112048 University of Porto, Rua Dr. Roberto Frias, Porto 4200-465, Portugal; 2 ALiCE − Associate Laboratory in Chemical Engineering, Faculty of Engineering, 112048 University of Porto, Rua Dr. Roberto Frias, Porto 4200-465, Portugal; 3 École polytechnique de l’Université de Lille, Cité Scientifique, Avenue Paul Langevin, Villeneuve d’Ascq Cedex 59655, France

## Abstract

This study demonstrates the successful immobilization of the enzyme glucose oxidase (GOx) onto 2D thermally exfoliated graphitic carbon nitride (GCN-T) via physical adsorption, producing a stable, reusable, and highly active bioconjugate. Binding was confirmed by scanning transmission electron microscopy (STEM) coupled with energy-dispersive spectroscopy (EDS), thermogravimetric analyses (TGAs), Fourier transform infrared spectroscopy (FTIR), and X-ray photoelectron spectroscopy (XPS). At the optimal conditions, GCN-T/GOx showed an immobilization yield of 88% and an activity of 0.54 U mg^–1^. Immobilized GOx retained 81% of its initial activity over 10 consecutive cycles and 50% after 10 storage/reuse cycles. While the free enzyme lost 27% of activity at 70 °C, the immobilized enzyme showed no activity loss after 2 h at the same temperature. Moreover, the 1.8-fold decrease in the Michalis–Menten kinetic constant indicates a better affinity with the substrate upon immobilization. As a proof of concept, GCN-T/GOx was used as a glucose sensor, showing a linear response from 2.5 to 25 mM, a sensitivity of 3.9 μA mM^–1^ cm^–2^, and a limit of detection (LOD) of 1 mM. The creation of the GCN-T/GOx bioconjugate is a breakthrough in sustainable solutions for the reusability and enhanced performance of enzymes, offering insights into the design of advanced biofunctional surfaces for nanotechnology, biosensing, biocatalysis, and beyond.

## Graphical abstract

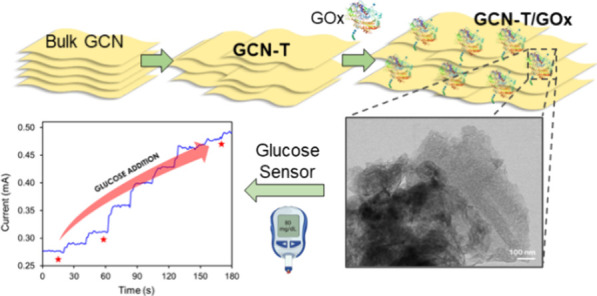

## Introduction

Enzyme immobilization is a well-known technique used in biotechnology and industrial processes, offering a range of significant advantages. Essentially, immobilizing an enzyme involves combining its kinetics, selectivity, and stability with the chemical and physical characteristics of the carrier to create a unique bioconjugate.^1^ This approach allows the reusability of the enzyme while reducing operational costs and waste.^2^ It also enables enzymes to work under a broader range of environmental conditions, including extreme pH and temperature, making them more versatile for various applications. From an industrial point of view, immobilization simplifies enzyme separation from the reaction medium, facilitating downstream processing and product purification and offering improved efficiency, sustainability, and cost-effectiveness.^3^ As a result, immobilization not only is a valuable technique for enzyme recovery but also has the potential to enhance the performance of the enzyme significantly. Physical adsorption is considered the most convenient method among the different immobilization strategies for its simplicity, affordability, and nonchemical bonding properties. Unlike covalent bonding or cross-linking, which may involve harsh conditions that can modify the enzyme’s active site, physical adsorption maintains the native structure and the activity of the enzyme, considering the reversible weak driving forces involved (van der Waals, electrostatic forces, hydrogen bonding, and ionic interactions).^4^ Although problems such as enzyme leakage, nonspecific binding, and the overloading of the support may occur, this method is particularly attractive in early-stage research for screening different immobilization conditions.

Glucose oxidase (GOx, E.C. 1.1.3.4) is an oxidoreductase enzyme that converts d-glucose into d-glucono-1,5-lactone and hydrogen peroxide (H_2_O_2_) using molecular oxygen as an electron acceptor.^5^ GOx is of high commercial value and is of increasing interest for various applications. However, several biological characteristics make it less suitable for industrial needs.^6^ Considering the numerous advantages, GOx has been immobilized onto several solid supports and used as glucose biosensors for diabetes monitoring,^7–9^ for cancer diagnosis and treatment,^10–14^ as biofuel cells,^15^ as food/beverage additives for prolonged shelf life,^16–18^ and in the textile industry for bleaching cellulose using the produced H_2_O_2_.^19^ Some examples of supports used for GOx in several applications include magnetic nanoparticles,^20–22^ cellulose membranes,^23^ porous silica,^7,24^ alginate-chitosan microcapsules,^25^ polymer microspheres,^26^ and gold nanoparticles.^27^

Carbon-based materials have also been extensively used as efficient support materials in enzyme immobilization.^28–30^ Their physicochemical properties, such as high surface area, excellent conductivity, and biocompatibility, make them ideal candidates for immobilizing enzymes. Moreover, the porous structure of carbon materials facilitates efficient enzyme loading, promoting the enhanced catalytic activity. Specifically, carbon nanotubes,^31^ graphene oxide,^32^ and graphene quantum dots^33^ have been used as platforms for the immobilization of GOx. However, as explained by Bolivar et al.,^34^ enzyme immobilization still needs to be fully explored, and there is always a margin for progression when taking full advantage of its properties.

Graphitic carbon nitride (GCN) is a 2D nanomaterial composed of covalently bonded earth-abundant elements (i.e., C, N, and H) and is the most stable allotrope among carbon nitrides. Until now, the focus has been on how well it performs in photo- and electrocatalysis, sensing, bioimaging, and energy conversion processes.^35–39^ However, due to its metal-free, nontoxic, and biocompatible features,^35,40^ together with its exceptional stability, ease of production, and tunable functionalization,^41–43^ GCN stands out as an exciting candidate for the immobilization of biomolecules. While Horseradish Peroxidase (HRP) has been successfully immobilized onto GCN for the photoassisted degradation of organic pollutants,^44–46^ to our knowledge, only one study has focused explicitly on the immobilization of GOx onto GCN for the amperometric glucose detection.^47^ Still, the screening of different immobilization conditions, kinetic studies, and the stability and reusability of the bioconjugate have never been determined.

In this work, GOx was immobilized onto thermally exfoliated GCN, referred to as GCN-T. Considering its simplicity and cost-effectiveness, physical adsorption was the selected method for immobilization. Here, GCN-T and the GCN-T/GOx bioconjugate were characterized, and different adsorption conditions (GOx concentration, pH, and immobilization time) were optimized to maximize the enzyme loading and activity. Moreover, the kinetic parameters were determined, and the bioconjugate’s thermal, operational, and storage stabilities were compared with the free enzyme. To the best of our knowledge, the immobilization parameters and stability tests have never been optimized for GOx adsorption onto GCN-T. As a proof of concept and to explore the practical applicability of the resulting optimal bioconjugate, GCN-T/GOx was studied for its capacity to monitor glucose. With this work, we aim to demonstrate whether GCN-T is a suitable and valuable immobilization platform for the immobilization of GOx and bring new insights into the field of knowledge regarding using this material in combination with other biomolecules.

## Experimental Section

### Materials and Reagents

Dicyandiamide (C_2_H_4_N_4_, 99%), horseradish peroxidase (HRP, ∼150 U mg^–1^), 4-aminoantipyrine (4-AAP), and Nafion (Nafion perfluorinated resin solution ∼5 wt %) were obtained from Sigma-Aldrich. High-purity glucose oxidase from *Aspergillus niger* (100 U mg^–1^), hydrogen peroxide (30 wt % in water), disodium hydrogen phosphate (≥99%), and phosphate buffer saline (PBS) were purchased from VWR International, LLC. TCI Chemicals supplied phenol (C_6_H_5_OH, ≥99%), and citric acid (≥99.5%) was supplied by Merck Chemical Company.

### Synthesis and Characterization of GCN

GCN was synthesized by the thermal decomposition of dicyandiamide in a Microwave Muffle Furnace Phoenix according to previously reported procedures.^48,49^ Dicyandiamide was put in a semiclosed crucible, and the temperature was increased to 450 °C and kept for 30 min. Afterward, the temperature was raised from 450 to 550 °C and maintained for 1 h. Both temperature ramps operated at 2 °C min^–1^. The resulting powder was crushed, washed, filtered, dried (80 °C overnight), and sieved (particle size <100 μm). Later, GCN was spread in open crucibles, and the temperature of the muffle furnace was increased to 500 °C and maintained for 2 h, thus producing an exfoliated material (GCN-T). The N_2_ adsorption isotherms of GCN and GCN-T were obtained at −196 °C in a Quantachrome Nova 4200e equipment. Furthermore, scanning transmission electron microscopy (STEM) images and energy-dispersive spectroscopy (EDS) mapping were obtained using a high-resolution HITACHI HD-2700 running at 200 kV. Fourier transform infrared (FTIR) measurements were performed on a JASCO FT/IT-6800 spectrometer coupled with a MIRacle Single Reflection attenuated total reflectance (ATR) accessory. Thermogravimetric analyses (TGAs) were performed using STA 490 PC/4/H Luxx Netzsch thermal equipment. In each test, ∼8 mg of the sample was placed on the crucible and heated from 50 to 800 °C (10 °C min^–1^) under air flow while the weight was continuously recorded. The surface chemical composition of the samples was studied by X-ray photoelectron spectroscopy (XPS). The analyses were performed in a Kratos Axis Ultra HSA spectrometer using an Al monochromator at 15 kV (90 W) in hybrid lens mode. The zeta potential was evaluated using a ZetaSizer Pro (Malvern Instruments, UK). The materials were diluted to a final concentration of 0.02 mg mL^–1^ in ultrapure water.

### GOx Activity Measurement

GOx activity was measured by a colorimetric method based on previously reported procedures.^7,20,23^ GOx catalyzes the oxidation of d-glucose to d-glucono-1,5-lactone and H_2_O_2_. With HRP, the produced H_2_O_2_ reacts with phenol and 4-AAP, resulting in a red/pink dye complex with a maximum adsorption at a wavelength of 510 nm. The activity of GOx was determined by performing two separate and sequential steps. In the first step, GOx (free or immobilized) reacted with 200 μL of 5 mM glucose solution. For the second step, the H_2_O_2_ released after the oxidation of glucose was quantified by removing 100 μL of the supernatant and adding it to an assay mixture containing 500 μL of the citrate/phosphate buffer (pH 6), 250 μL of 0.4 mM 4-AAP, 100 μL of 40 mM phenol, and 10 U of HRP. The absorbance of the resultant pink quinoneimine complex determined spectrophotometrically (OceanOptics USB2000+ UV–vis spectrophotometer) was translated to the H_2_O_2_ concentration through a calibration curve previously plotted. One unit of GOx activity is equivalent to the amount of enzyme that produces 1 μmol of H_2_O_2_ per minute at pH 6 and 25 °C. The linearity of the reaction was verified and established as 4 min. The performance of two separate steps to calculate the enzymatic activity is essential; otherwise, the adsorption of the pink complex on the GCN-T could result in an underestimation of the actual activity. All the experiments were performed in duplicate. The immobilization yield (%) is calculated by dividing the activity of the free enzyme remaining in the supernatant after immobilization by the activity of the free enzyme before immobilization.

### Optimization of GOx Immobilization onto GCN-T

The immobilization of GOx by physical adsorption was studied by adding 200 μL of GOx solution, prepared in the citrate/phosphate buffer, to 2 mg of GCN-T. The immobilization was performed by stirring the mixtures at 50 rpm in a vertical mini rotator (model PTR-35). The experimental conditions (GOx concentration, pH, and immobilization time) were studied and optimized to maximize the activity of GOx and immobilization yield. Different enzyme concentrations, 0.1–1 g L^–1^ (10 a 100 U mL^–1^), were tested to determine the materials’ adsorption capacity. The Langmuir isotherm was used to determine the adsorption equilibrium behavior of GOx. The influence of pH was evaluated by preparing the initial enzyme solution in different citrate/phosphate buffers (50 mM) from pH 2 to 9. The contact time was optimized at the optimal pH by studying the immobilization at different time intervals at room temperature.

### GOx Kinetics

The kinetic parameters (Michalis–Menten), *K*_m_ and *v*_max_, of free GOx and GCN-T/GOx were calculated by determining the initial rates of glucose oxidation (0.1–20 mM) by GOx (0.5 g L^–1^) prepared in the citrate/phosphate buffer (pH 6) at 25 °C. The Michaelis–Menten kinetics describes the rate of enzymatic reactions by relating the reaction rate to the substrate concentration (eq 1), where *v*_max_ is the maximum reaction rate when the enzyme is saturated with substrate and *K*_m_ represents the substrate concentration at which the reaction rate is half of *v*_max_.

$$v=\frac{v_{\max}[S]}{K_{m}+[S]}$$ 1

### Bioconjugate GCN-T/GOx Stability

Two strategies were adopted to evaluate the reusability of the bioconjugate. After the first use, GCN-T/GOx was washed with the citrate/phosphate buffer (pH 6). Then, the buffer was removed, and fresh glucose solution was added to the bioconjugate, which started the next reusability cycle. In the first strategy, the bioconjugate was stored at 4 °C for 24 h between cycles. The bioconjugate was reused immediately after washing (without storage) in the second strategy. These steps were repeated 10 times. The activity of cycle 1 was defined as 100%. To determine the storage stability, the activity of free and immobilized GOx was measured over 1 month of storage at 4 °C. The immobilized enzyme was stored in 1 mL of the citrate/phosphate buffer (pH 6). The initial activity was considered 100%, and the relative activities were calculated by comparison. The thermal stability of free GOx and GCN-T/GOx (prepared with the citrate/phosphate buffer (pH 6)) was determined by monitoring the residual activity of the enzyme after a 4 h exposure to different temperatures (37–80 °C). Aliquots of the reacting solutions were taken every hour and analyzed for enzymatic activity as described above.

### Fabrication of the FTO/Nf/GCN-T/GOx Electrode and Photoelectrochemical Experiments

A fluorine-doped tin oxide (FTO) coated glass was used to support the working electrode. GCN-T/GOx (10 mg) was suspended in 1.25 mL of distilled water and 100 μL of 5 wt % Nafion (Nf) based on previously reported methods.^50,51^ The suspension (540 μL; ∼4 mg of GCN-T/GOx) was spread onto a 2 cm^2^ area of the FTO glass by drop-casting. The prepared electrodes (FTO/Nf/GCN-T/GOx) were heated at 50 °C for 1 h and then dried at 4 °C. For comparison, an electrode without GOx was fabricated using a similar method. Photoelectrochemical studies were executed in a Zahner Zennium PRO electrochemical workstation with a PP-211 external potentiostat. A three-electrode cell configuration, composed of a working, a counter (platinum wire), and a reference electrode (Ag/AgCl, 3 M KCl), was used for photoelectrochemical characterization. Cyclic voltammetry studies were performed in 50 mL of 0.15 M PBS (pH 7.4) from −1 to 2 V at a scan rate of 100 mV s^–1^. The amperometric current–time response analysis was performed under room temperature (∼20 °C) by successively adding glucose solution (2.5–35 mM) into a continuously stirred 0.15 M PBS (pH 7.4) solution at an applied potential of 1.75 V (vs Ag/AgCl).

## Results and Discussion

### Characterization of the Support and Bioconjugate

The thermal treatment of bulk GCN promoted layer splitting and layer-by-layer thermal oxidation of the material.^52,53^ This exfoliation resulted in a 14-fold increase in the Brunauer–Emmett–Teller specific surface area (*S*_BET_) of GCN-T when compared to GCN, which is consistent with previous findings.^48,49,54^ As previously reported, thermal exfoliation introduces surface defects such as terminal amines, which create more anchoring points that could favor enzyme bending. However, no oxygen defects are introduced upon the second thermal treatment considering that the oxygen content remains very low in GCN and GCN-T (<2.1%).^55^

This increase in surface area is an asset, as it enhanced the efficiency of biomolecule immobilization onto solid supports.^56^ Our research group has widely studied the characterization of GCN and GCN-T over the past few years.^48,49,57^ Therefore, only the most relevant properties intended for the proposed application are highlighted here.

STEM images and EDS elemental mapping of GCN-T and the GCN-T/GOx bioconjugate were acquired to study the morphology of the materials and confirm the immobilization of GOx, respectively (Figure 1). Figure 1a shows a material composed of well-spread thin layers that resulted from the thermal exfoliation of the stacked GCN bulk. By comparing the morphology of GCN-T with the GCN-T/GOx bioconjugate (Figure 1b), it is still possible to see the characteristic layer splitting of the material together with some aggregated sections. Comparing the C, N, and O content of GCN-T (Figure 1c,e,g, respectively) with GCN-T/GOx (Figure 1d,f,h, respectively), it is possible to see that C is evenly distributed in both surfaces. In contrast, the N and O content increased with the adsorption of GOx due to the presence of characteristic amine, amide, and carboxyl groups.^58^ The residual amount of O on the surface of GCN-T can be attributed to its contact with the external environment.

**Figure 1:**
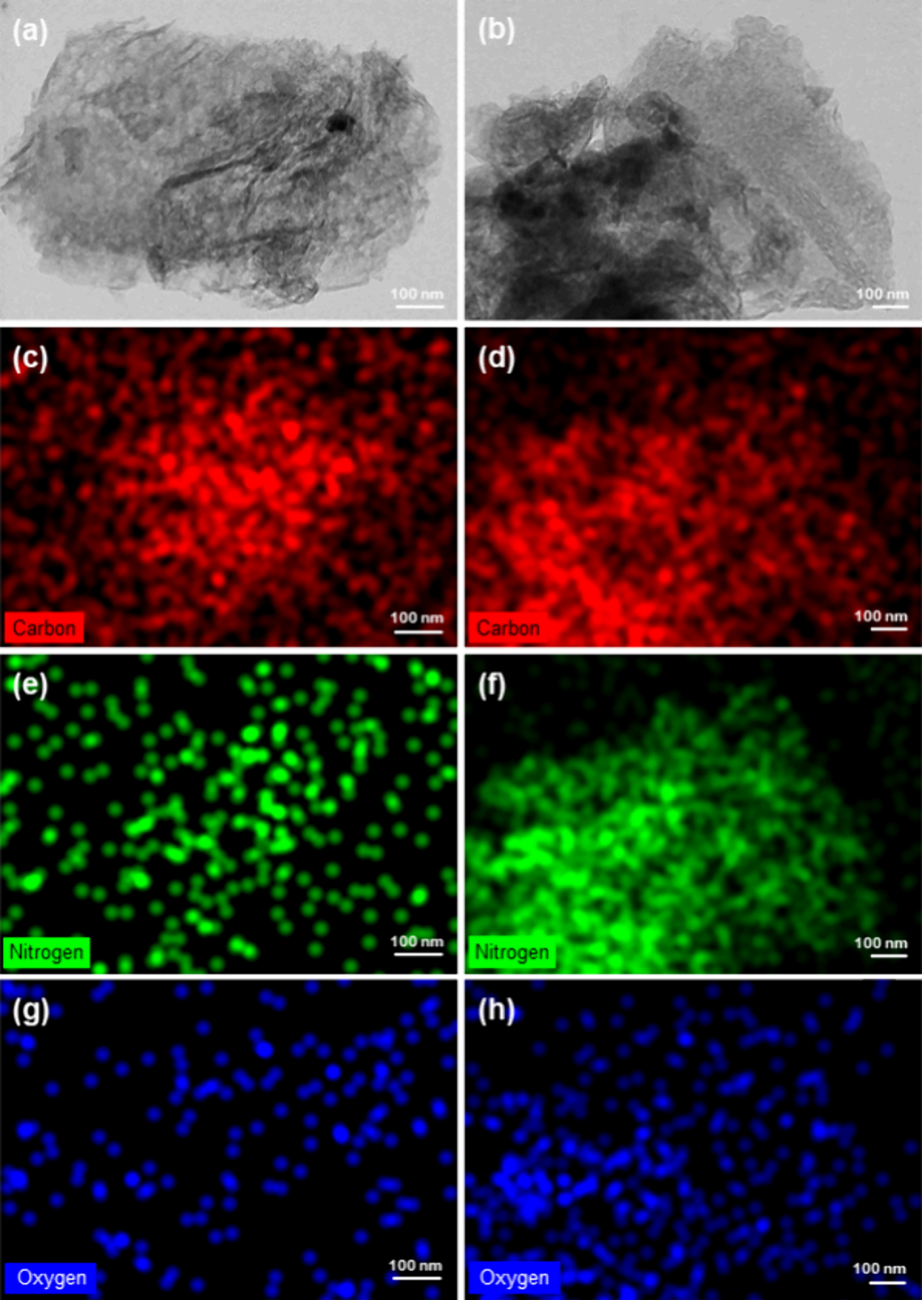
STEM micrographs of GCN-T (a) and the GCN-T/GOx bioconjugate (b). Corresponding elemental EDS mapping images of C, N, and O elements present before (c, e, g) and after (d, f, h) immobilization of GOx onto GCN-T.

We also aimed to verify the immobilization of GOx onto GCN-T via TGA. As shown in Figure 2a, weight loss was negligible up to ca. 550 °C in the thermogram of GCN-T. Above 715 °C, the carbon material was fully gasified. Conversely, the TGA profile of the GCN-T/GOx bioconjugate shows its first weight loss at about 300 °C related to the thermal degradation of the enzyme. The simultaneous pyrolysis of GOx and GCN-T is translated in the second weight loss, which starts at 500 °C. At about 720 °C, a plateau is reached with the complete pyrolysis of the bioconjugate. The surface functional groups of GCN-T and GCN-T/GOx were investigated by ATR-FTIR analysis to confirm the physical adsorption of GOx on GCN-T (Figure 2b). The GCN-T spectrum shows the characteristic peak of tri-s-triazine units at around 804 cm^–1^ and the typical stretching of C=N and CO– by the peaks at 1225, 1312, 1396, 1536, and 1625 cm^–1^.^59,60^ The representative IR peaks of GOx are the amide I band (around 1645 cm^–1^) and amide II band (around 1530 cm^–1^), which are visible in the presented spectrum. Compared with GCN-T, some differences appeared in the FTIR spectrum of GCN-T/GOx, with the intensities decreasing. Lower intensities of the peaks between 1227 and 1624 cm^–1^ are associated with the aromatic C–N and C–N skeleton, which may be related to the strong interaction of the enzyme with the GCN-T surface.^61,62^ Moreover, the XPS spectrum analysis provides significant insights into the changes in the surface chemical composition of GCN-T and its bioconjugate with GOx. The full scan of the materials is shown in Figure S1 in the Supporting Information, while the high-resolution peaks of C 1s, N 1s, and O 1s are displayed in Figure S2. In the case of GCN-T, similarly to what has been previously reported,^49^ four main peaks were identified. A C1 peak at 285.0 eV was observed, corresponding to the C­(−N)_3_ planar trigonal carbon geometry of GCN.^63^ The C2 peak at 286.4 eV is associated with sp^3^-bonded defects on the GCN-T surface, likely from amino functional groups (C–NH_2_).^64,65^ The C3 peak at 288.5 eV represents the dominant carbon environment in the catalyst, indicative of sp^2^-bonded carbon in N-containing aromatic rings (N–C=N).^66^ Finally, a C4 weak peak at 293.8 eV is attributed to π electron delocalization in the GCN-T heterocycles.^63^ Upon immobilization of GOx, the C 1s spectra show a notable reduction in the C1 peak to 14.99%, indicating an alteration in the carbon structure, likely due to the interaction of the enzyme with the surface, which disrupts the C–C/C=C bonds. This decrease suggests that the immobilization of GOx affects the sp^2^-hybridized carbon network. There is also an increase in the C2 peak to 9.67%, which corresponds to C–N or C=N bonds, likely formed due to interactions between the enzyme’s amino groups and the GCN-T surface. Furthermore, the C3 peak increases to 72.14%, indicating that N–C=N bonds become more prevalent, possibly due to additional nitrogen-containing groups from GOx. The C4 peak rises slightly to 3.20%, possibly associated with additional C=O groups, perhaps arising from the enzyme or enzyme–surface interactions. Furthermore, the minimal changes in pyridinic nitrogen (N3) suggest that the core structure of GCN-T remains largely intact, while the surface interactions with GOx modify the surface chemical composition, particularly with regard to carbon and nitrogen. The increase in oxygen species (O2) further supports the presence of GOx on the surface, highlighting the successful enzyme immobilization process.^67^

**Figure 2:**
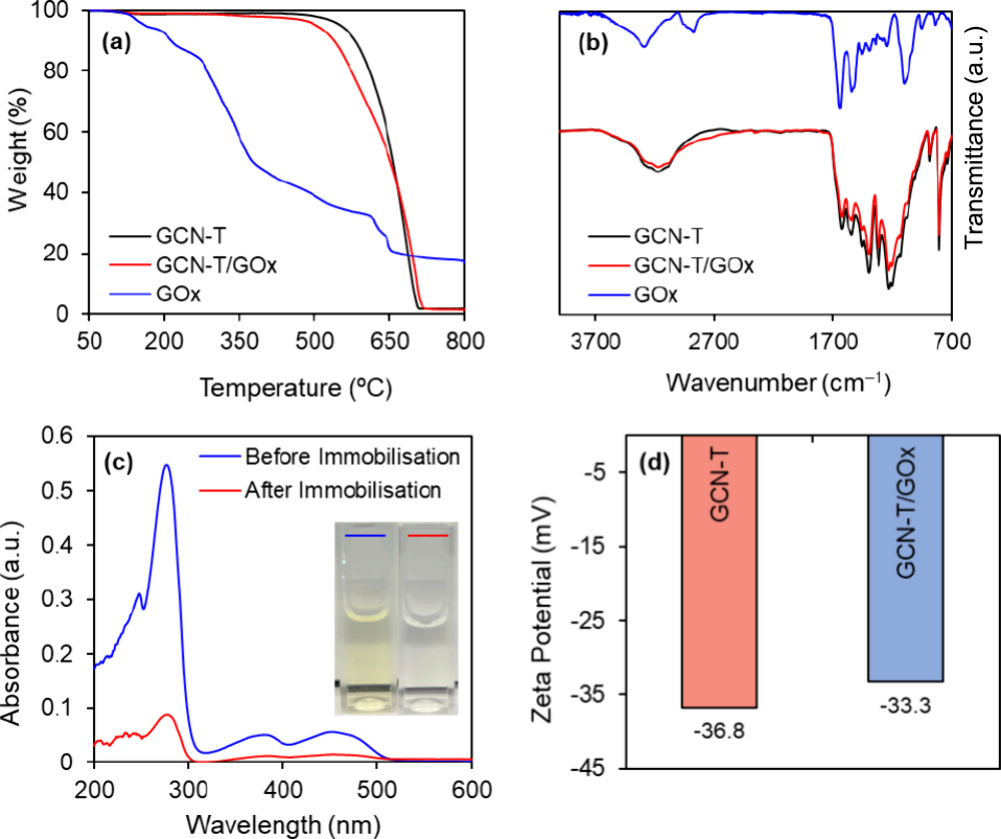
TGA thermograms (a) and ATR-FTIR spectra (b) of GCN-T, GCN-T/GOx, and GOx. (c) UV–vis absorbance spectra of GOx solution before (blue) and after (red) immobilization onto GCN-T (inset image showing the respective color change). (d) Zeta potential measurements of GCN-T and GCN-T/GOx.

UV–vis spectra were obtained to compare the glucose solution before and after immobilization (Figure 2c). Before immobilization, a strong absorbance peak is observed at 280 nm characteristic of the polypeptide chains, while the weak peaks around 386 and 458 nm represent the oxidized form of flavin groups in the protein structure.^68^ After immobilization, the position and shape of the absorption bands are the same as those of free GOx but with a significant decrease in intensity, which is also confirmed through the color change of the solution (Figure 2c; inset image). The zeta potential measurements of GCN-T and GCN-T/GOx were also performed and are represented in Figure 2d. The observed decrease in the magnitude of the zeta potential (from −36.8 to −33.3 mV) upon immobilization indicates a successful modification of the material′s surface charge. This change is consistent with findings in the literature. For instance, Rusu et al.^69^ reported that the interaction between GOx and magnetic nanoparticles functionalized with succinic anhydride led to a shift in the surface charge, as evidenced by zeta potential measurements. This shift was attributed to the adsorption of the enzyme onto the nanoparticle surface, modifying its charge properties.

### Immobilization of GOx onto GCN-T

The efficiency of the immobilization of GOx onto GCN-T was analyzed through the balance between the activity of the bioconjugate and the immobilization yield. For each studied condition, the maximum activities achieved were considered 100%, and the results were presented as relative values.

The enzyme/support mass ratio was investigated to study the adsorption capacity of GCN-T. GOx was immobilized onto GCN-T at pH 6 for 60 min using different enzyme concentrations (from 0.01 to 1 g L^–1^) while keeping the mass of GCN-T at 2 mg. The results, shown in Figure 3a, indicate a steady increase in the activity of the bioconjugate with the GOx concentration, starting to stabilize at 0.5 g L^–1^. Unlike the activity, there is almost a total adsorption of GOx (immobilization yields >80%) until 0.3 g L^–1^. From this point onward, there is a gradual decrease in the immobilization yield, reaching 43% when immobilizing 1 g L^–1^ of GOx. This behavior suggests that for concentrations below 0.3 g L^–1^, the GCN-T can adsorb the total amount of GOx molecules available. However, at 0.5 g L^–1^, GCN-T has already achieved its maximum capacity for GOx adsorption.

**Figure 3:**
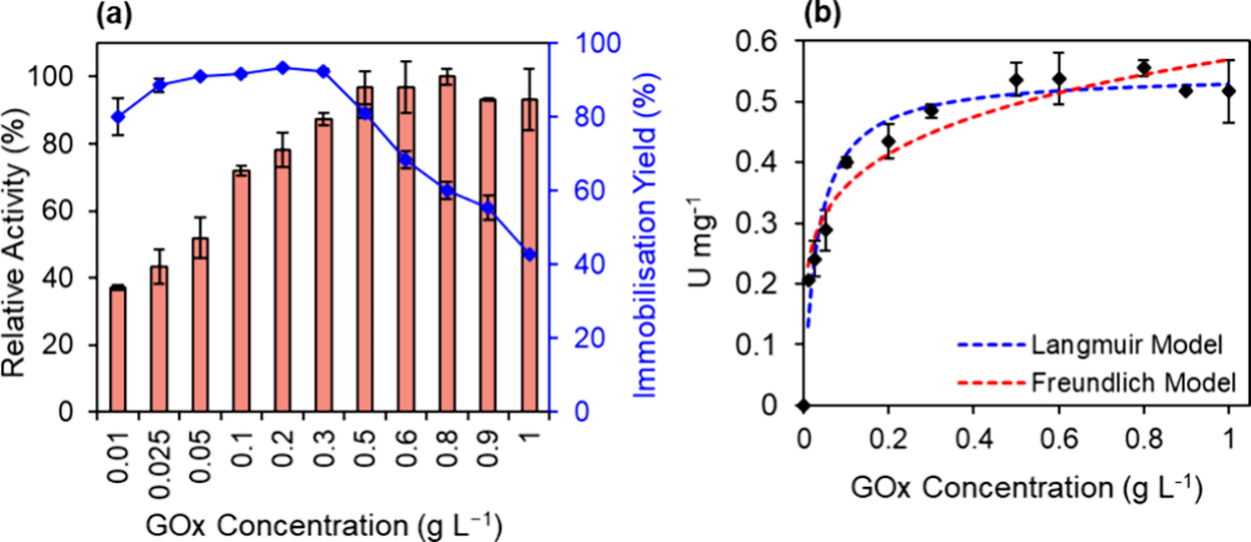
(a) Influence of GOx concentration on the activity (columns) and immobilization yield (symbols, line). (b) Langmuir and Freundlich isotherm models for the adsorption of GOx onto GCN-T. Conditions: 2 mg of GCN-T, pH 6, and 60 min of contact time.

To further investigate the interaction between GOx and GCN-T, we determined the adsorption isotherm. The Langmuir model assumes monolayer adsorption on a homogeneous surface, leading to a saturation point, where a maximum adsorption capacity is reached. In contrast, the Freundlich isotherm model applies to heterogeneous surfaces, allowing multilayer adsorption without a defined maximum. As seen in Figure 3b, the experimental data were better adjusted by the Langmuir (*R* ^2^ of 0.9878) than the Freundlich model (*R* ^2^ of 0.9595), suggesting monolayer adsorption of GOx on the support surface. Based on Langmuir isotherm parameters, GCN-T has a maximum adsorption capacity (*q*_max_) for GOx of 0.54 U mg^–1^, with an adsorption equilibrium constant (*K*) of 31 L g^–1^. Given that commercial GOx contains 100 U/mg, a loading capacity of 54 mg of GOx per gram of GCN-T was achieved. With a balance between (0.54 U mg^–1^) and an immobilization yield of 81%, a GOx concentration of 0.5 g L^–1^ was selected for further experiments. With a balance between (0.54 U mg^–1^) and an immobilization yield of 81%, a GOx concentration of 0.5 g L^–1^ was selected for further experiments.

The influence of pH was also studied to understand better the driving forces of interaction between GOx and GCN-T. Physical adsorption is the most simple, fast, and cost-effective method of enzyme immobilization and is characterized by the formation of reversible surface interactions (noncovalent bonds). Due to the weak driving forces and the absence of chemical modifications, this technique does not affect the native structure of the enzyme and enables it to retain its activity.^70^ In this sense, a wide pH range was studied (between 2 and 9) to immobilize 0.5 g L^–1^ of GOx onto GCN-T for 60 min. According to Figure 4a, the bioconjugate strongly prefers acidic environments, especially between 4 and 6. The isoelectric point of GOx is 4.2,^71^ and the *pH*_PZC_ of GCN-T is 6;^72^ between these values, the enzyme and the support have opposite charges, contributing to the formation of electrostatic interactions. Still, considering the maximum activity achieved for pH 6 and an immobilization yield of 90%, pH 6 was chosen as the optimal pH for further experiments. Similar results were obtained when immobilizing GOx onto chitosan-coated Fe_3_O_4_ nanoparticles^73^ and tannic-acid-modified CoFe_2_O_4_ magnetic nanoparticles.^74^ In addition to the pH optimization study for the immobilized enzyme, we also evaluated the activity of the enzyme in its free form at different pH values, observing similar stability profiles. This result indicates that pH variations do not compromise the structural integrity of the enzyme, further supporting the reliability of our immobilization approach.

**Figure 4:**
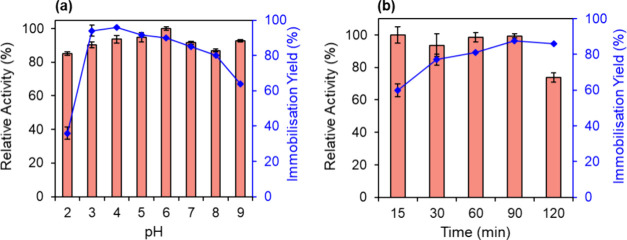
(a) Influence of pH on the activity (columns) and immobilization yield (symbols, line) for 60 min of contact time. (b) Effect of contact time on the activity (columns) and immobilization yield (symbols, line) at pH 6. Set conditions: 0.5 g L^–1^ of GOx immobilized onto 2 mg of GCN-T.

The influence of the immobilization time was also studied to maximize the immobilization yield and activity of the bioconjugate. In this sense, the adsorption of GOx onto GCN-T was studied for five different time intervals, between 15 and 120 min, using 0.5 g L^–1^ of GOx at pH 6. The impact of contact time on the bioconjugate′s activity and immobilization yield is represented in Figure 4b. Overall, with time, the immobilization yield increases, and the activity of the bioconjugate decreases. A low immobilization yield (around 60%) was observed for a 15 min period, while a high activity was reported. Lower amounts of the enzyme loaded onto the support will allow the enzyme to easily react with the substrate by avoiding mass transfer limitations that could arise from higher enzyme loadings. Substrate molecules may not be able to reach GOx’s active site as more enzyme is immobilized due to layer stacking of adsorbed GOx and uncontrolled accumulation of enzyme.^75^ After 30 min of contact between the enzyme and GCN-T, an increase in the immobilization yield was observed, reaching its maximum after 90 min. Regarding the activity, there was a decrease from 90 to 120 min of contact time, suggesting that the enzyme was already completely immobilized. Considering the relative activity of 99% and immobilization yield of 88%, 90 min was chosen for further experiments.

### GOx Kinetics

The effect of glucose concentration (0.1–20 mM) on the glucose oxidation rate by free GOx and the GCN-T/GOx bioconjugate was evaluated through the Michaelis–Menten equation. The kinetic constants, *K*_m_ and *v*_max_, for free and immobilized GOx are displayed in Table 1, together with the turnover number (*k*_cat_) and the corresponding catalytic efficiency (*k*_cat_ */K*_m_). *K*_m_ is a measure of the enzyme’s affinity to the substrate: the lower the *K*_m_ is, the higher is the enzyme’s affinity to the substrate. Meanwhile, *k*_cat_ is the maximum number of substrate molecules converted to product per active site of the enzyme per unit time. A higher *k*_cat_ value means that the enzyme can convert the substrate to the product more quickly. Mathematically, it corresponds to *v*_max_ divided by the enzyme concentration (0.5 g L^–1^).

**Table 1:** Michaelis–Menten Kinetic Parameters for Free GOx and the GCN-T/GOx Bioconjugate

	free GOx	GCN-T/GOx
*K*_m_ (mM)	2.1	1.5
*v*_max_ (mM min^–1^)	0.65	0.66
*k*_cat_ (min^–1^)	209	213
*k*_cat_ */K*_m_ (mM^–1^ min^–1^)	100	140

The free enzyme and GCN-T/GOx bioconjugate showed *K*_m_ values of 2.1 and 1.5 mM and *k*_cat_ values of 209 and 213 min^–1^, respectively. These results suggest a 1.4-fold increase in the immobilized enzyme affinity for the substrate and a quicker conversion of glucose. The enzyme’s increased substrate affinity could be attributed to a favorable modification in the enzyme’s structural organization induced by the immobilization process.^76^ Moreover, the similar *v*_max_ values obtained for free and immobilized GOx suggest minimal mass transfer limitations and that the active site remains accessible after immobilization.^20^ The calculated kinetic parameters translate into a higher catalytic efficiency (*k*_cat_ */K*_m_) of the immobilized GOx compared to the free GOx (140 and 100 mM^–1^ min^–1^, respectively).

Similar results were obtained by Kouassi et al.^20^ when immobilizing glucose oxidase onto functionalized magnetic nanoparticles, reporting a 1.8-fold decrease in *K*_m_ and higher *v*_max_ values of the immobilized enzyme when compared with free GOx. However, the same did not happen when graphene oxide was used as a support for GOx,^32^ resulting in an increase in the *K*_m_ value upon immobilization and a decrease in the *v*_max_. These findings suggest that the enzyme might have suffered conformational changes during immobilization, and the support limited the access of GOx to the substrate.^77^

### Reusability and Storage Stability

One of the main benefits of the immobilized enzyme over its native form is its operational stability, a crucial factor for the cost-effective use of the enzyme in commercial applications. Immobilized enzymes can be recovered and reused after each step, while free enzymes are limited to a single use. The operational stability of the GCN-T/GOx bioconjugate was evaluated throughout 10 cycles of glucose oxidation. When the bioconjugate was reused immediately after the washing step, there was almost no activity loss over the cycles, retaining 81% of its initial activity after the 10 cycles. As reported in several works, the reusability test was also explored in combination with 24 h storage between cycles.^78,79^ In this case, the immobilized enzyme exhibited an excellent performance up to 5 reuses and 50% of its original activity after 10 reuses. The results are shown in Figure 5a. Even though other studies have reported higher loading of GOx onto the support material,^7,20,21,80^ GCN-T/GOx presented a very high reusability capacity compared to other used immobilization platforms. For instance, immobilized GOx onto Fe_3_O_4_/SiO_2_ magnetic nanoparticles could only retain 60% of initial activity after six consecutive operations. This activity loss might be related to the successive washing steps and some conformational changes that can occur upon immobilization.^21^ Even in studies employing cross-linking^81^ and entrapment^82^ for the immobilization of GOx, the bioconjugates retained 57 and 50% of activity, respectively, after seven cycles. This work proved the effectiveness and simplicity of physical adsorption compared to other methods that involve multiple chemicals and complex procedures, which are often challenging and time-consuming and may damage the enzyme structure. This natural affinity between GCN-T and GOx is definitely a very valuable and promising property compared to other supports. Storage stability is also essential when studying the efficiency of enzyme immobilization onto a support. The storage stabilities of free and immobilized GOx were evaluated for 28 days at 4 °C and are presented in Figure 5b. After 2 weeks of storage, the bioconjugate retained 89% of its initial activity. After 1 month, this value decreased to 82%. Throughout this time, the activity of GCN-T/GOx was always very similar to that of free GOx, meaning that the immobilization process did not affect the stability of the enzyme. As previously demonstrated, the immobilization onto GCN-T allows GOx to maintain its catalytic activity while allowing the enzyme to be reused. Similar results were obtained by Huang et al.,^21^ showing that GOx covalently bonded onto magnetic nanoparticles retained 75% of its initial activity after 1 month of storage at 4 °C. These findings highlight the promising properties of GCN-T as support for GOx as physical adsorption interactions were enough to ensure the stability of the bioconjugate.

**Figure 5:**
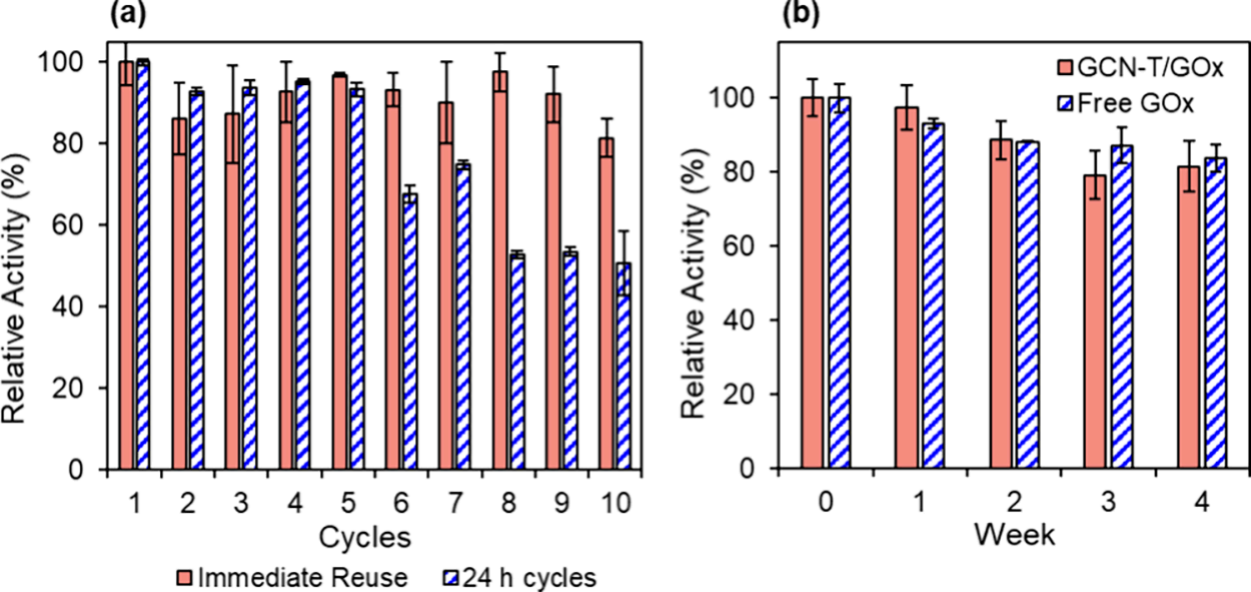
(a) Reusability of the GCN-T/GOx bioconjugate over 10 cycles. Solid columns represent the activity of the bioconjugate over immediate reuse. The dashed columns represent the activity of the bioconjugate over 24 h cycles. (b) Storage stability of the GCN-T/GOx bioconjugate (solid columns) and free GOx (dashed columns) at 4 °C.

### Thermal Stability

It is often anticipated that immobilization will increase the enzyme’s tolerance to a broader range of temperatures, working as a barrier against the enzyme’s denaturation or abrupt conformational changes.^83^ However, when the immobilization is performed by physical adsorption, temperature can promote the desorption of the enzyme from the material. The effect of temperature on free and immobilized GOx activity was examined by measuring its relative activities after incubation at different temperatures. From Figure 6, it can be observed that from 37 to 60 °C, both the free and the immobilized enzymes retained their initial activity after 2 h of incubation. Nonetheless, after 2 h at 70 °C, the activity of the free enzyme decreased to 73%, while the immobilized enzyme showed no activity loss. This significant improvement against harsh environments might be related to the diffusional limitations of the immobilized enzyme molecule.^76^ Total loss of activity occurred at 80 °C for free GOx and the GCN-T/GOx bioconjugate due to the denaturation of the enzyme. According to Rauf et al.,^23^ GOx immobilized on a cellulose acetate-poly­(methyl methacrylate) membrane performed better in terms of thermal stability than the free enzyme. The immobilized enzyme retained 46% of the original activity at 70 °C, while the free enzyme activity decreased drastically beyond 55 °C. However, GOx was immobilized in this case through the glutaraldehyde cross-linking method, promoting the formation of stronger bonds between the enzyme and the material when compared to the physical adsorption employed in the present work.

**Figure 6:**
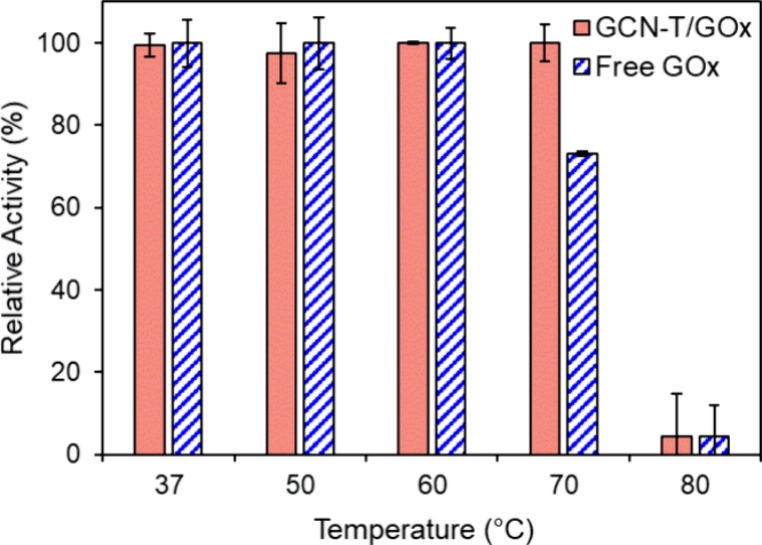
Thermal stability of the GCN-T/GOx bioconjugate (solid columns) and free GOx (dashed columns). Activity measured after 2 h of incubation.

### GCN-T/GOx as a Glucose Sensor

Glucose electrochemical sensors have been extensively studied and fabricated for biological analysis, food processing, environmental monitoring, and clinical detection.^84^ GOx is the model enzyme used for the enzymatic determination of glucose.^85^ Considering the outstanding stability and high catalytic activity of GCN-T/GOx, this bioconjugate was tested for its sensing capacity to monitor glucose. For this, GCN-T/GOx was deposited onto an FTO glass along with Nafion, as presented in Figure 7. The prepared electrodes’ electrocatalytic activity and amperometric response were studied.

**Figure 7:**
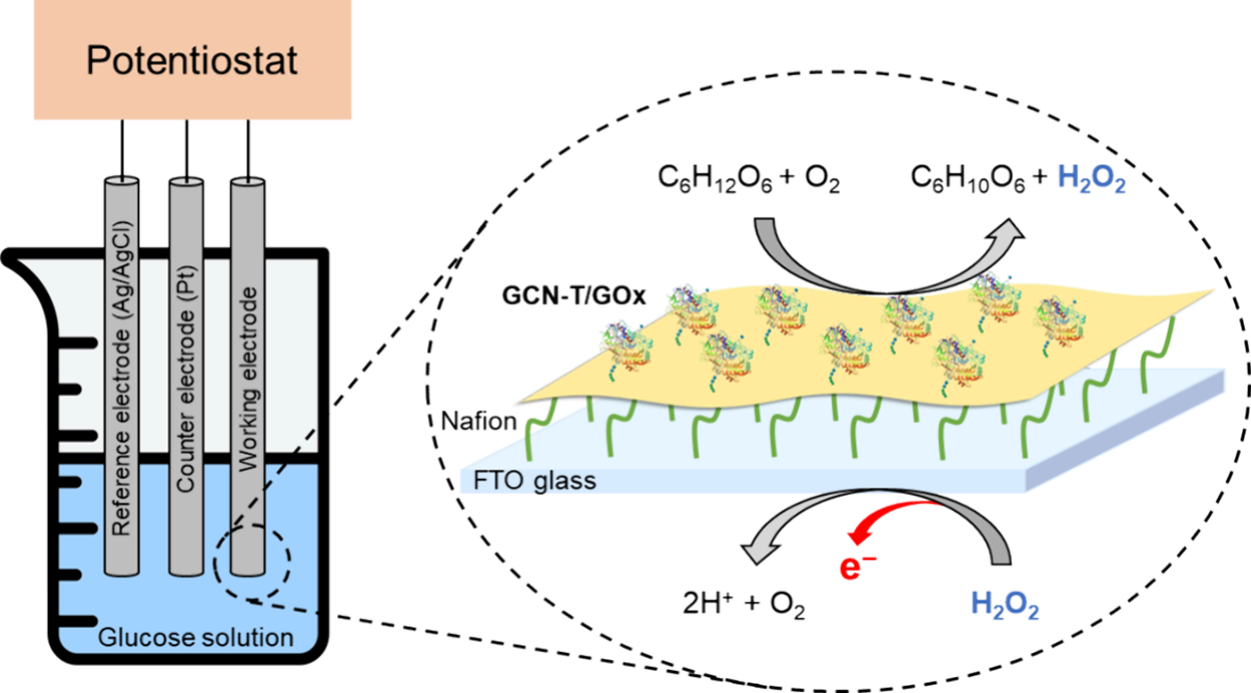
Schematic representation of the FTO/Nf/GCN-T/GOx electrode fabrication.

### Electrocatalytic Activity of FTO/Nf/GCN-T/GOx

The performance of the prepared electrode was analyzed by cyclic voltammetry (CV). Figure 8 presents the CV signals of FTO/Nf/GCN-T and FTO/Nf/GCN-T/GOx (with and without glucose) in 0.15 M PBS (pH 7.4). The CV curves indicate that the integration of GOx onto the working electrode did not result in any increase in the current increase. However, when glucose was added, both the oxidation and reduction peaks increased, reflecting the electron transfer between the active sites of GOx and glucose. CV at different scan rates was also performed and is presented in Figure S3, showing that the anodic and cathodic peak currents increase linearly with an increase in the scan rate. Additional CV measurements were also performed with varying glucose concentrations to show the linearity in detection and identify the oxidation and reduction peaks associated with the catalytic reaction (Figure S4).

**Figure 8:**
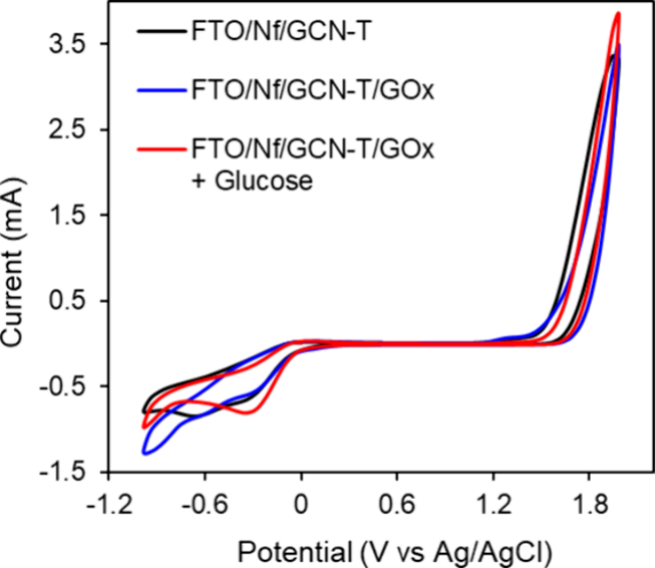
Cyclic voltammograms of FTO/Nf/GCN-T and FTO/Nf/GCN-T/GOx (without glucose and in the presence of 5 mM) in 0.15 M PBS (pH 7.4) at a scan rate of 100 mV s^–1^.

### Amperometric Detection of Glucose

Chronoamperometric measurements were recorded to determine the limit of detection (LOD), linear range, and sensitivity for glucose detection at FTO/Nf/GCN-T/GOx. Figure 9a displays a typical amperometric current–time response of the FTO/Nf/GCN-T/GOx upon the successive addition of glucose (2.5–35 mM) in continuously stirred 0.15 M PBS (pH 7.4). Glucose concentration variations were monitored by the oxidation current of H_2_O_2_ generated by the enzymatic reaction.^47^ Upon glucose addition, an increase in the oxidation current was observed, with a rapid amperometric response time averaging under 5 s. The corresponding calibration curve (Figure 9b) shows a linear response from 2.5 to 25 mM, with a high correlation coefficient (*R* ^2^ = 0.9951) and a sensitivity of 7.8 μA mM^–1^ (3.9 μA mM^–1^ cm^–2^). Based on a signal-to-noise ratio (S/N) of 3, the LOD was estimated at 1 mM.

**Figure 9:**
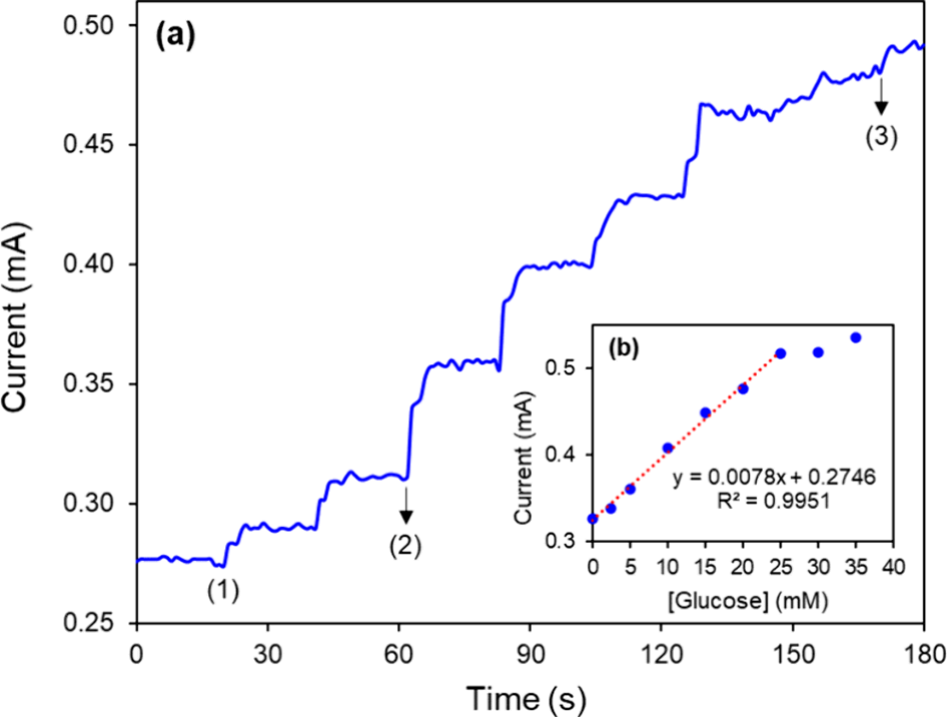
(a) Amperometric response of the FTO/Nf/GCN-T/GOx with successive glucose additions into 0.15 M PBS buffer solution (pH 7.4) (1 → 2, 2.5 mM steps; 2 → 3, 5 mM steps). (b) Calibration curve of the FTO/Nf/GCN-T/GOx. The applied potential was 1.75 V (vs Ag/AgCl).

Table 2 summarizes the electrochemical behavior (linear range, LOD, and sensitivity) of some of the most recent carbon-based modified electrodes reported for glucose sensing, such as single-walled carbon nanotubes (SWCNT-GOx),^86^ N-doped carbon nanospheres (CNS-GOx),^87^ electrochemical reduced graphene oxide-poly­(l-lysine) (ERGO-PLL),^88^ 3D N-doped CNTs supported by a carbon foam (N-CNT@CF),^89^ 3D graphene thin film (3DG-GOD),^90^ graphene quantum dot-luminol-Ag nanoparticles (GQD-luminol-AgNP),^91^ and 2D graphitic carbon nitride (g-C_3_N_4_) nanosheets.^47^ All of these results demonstrate the effective use of various carbon nanostructures and glucose oxidase for glucose monitoring, showcasing the wide range of detectable concentrations and sensitivity limitations for different practical applications. According to the World Health Organization, the expected values for normal fasting blood glucose concentration are between 70 (3.9 mM) and 100 mg dL^–1^ (5.6 mM). If blood glucose is higher than 126 mg dL^–1^ (7 mM), then diabetes can be diagnosed. Considering these theoretical values, the GCN-T/GOx biosensor developed in this work shows a linear range and a suitable LOD for detecting blood glucose. Meanwhile, the relatively low sensitivity of the developed glucose biosensor can be attributed to the immobilization of GOx via physical adsorption. Although this approach is sufficient to achieve efficient enzyme attachment, the weak interactions between the enzyme and the material may result in limited electron transfer efficiency and reduced surface coverage compared to covalent bonding methods. Nevertheless, GCN-T offers potential advantages, including simpler and cost-effective synthesis methods. Considering the obtained results, further optimization could be explored by modifying the surface of GCN-T with functional groups (e.g., amino, carboxyl) to enhance enzyme binding strength and orientation. This immobilization could also be extended to other oxidase enzymes (e.g., cholesterol oxidase, alcohol oxidase, and lactate oxidase), enabling the biosensor to detect a wider range of substrates.

**Table 2:** Comparison between Different Carbon-Based Electrodes Employing GOx for Glucose Monitoring

electrode	linear range	LOD	sensitivity (μA mM^–1^ cm^–2^)	ref
SWCNT-GOx	1–100 mM	10 μM	9.15	^86^
CNS-GOx	0.08–2.04 mM	39.1 μM	7.31	^87^
ERGO-PLL	0.005–9 mM	0.002 μM	8.00	^88^
GOD/N-CNT@CF	0.05–15.55 mM	5 μM	15.87	^89^
3DG-GOD	0.3–6 mM	0.2 mM	1.63	^90^
GQD-luminol-AgNP/GOx	25–250 μM	8 μM		^91^
GOx/g-C_3_N_4_	50 μM–2 mM	5 μM	21.70	^47^
GCN-T/GOx	2.5–25 mM	1 mM	3.90	this work

## Conclusions

This study successfully established a straightforward and cost-effective method for immobilizing glucose oxidase (GOx) onto GCN-T through physical adsorption. The optimization of enzyme loading, pH, and contact time significantly enhanced the immobilization efficiency and the resulting bioconjugate’s performance. Under the optimal conditions (0.5 g L^–1^ of GOx, pH 6, and 90 min of contact time), the enzyme's affinity toward glucose increased, and the bioconjugate was more stable than free GOx. Even when reusability was combined with 24 h storage, the bioconjugate showed almost no activity loss after five cycles compared to other reported supports. After a month of storage at 4 °C, GCN-T/GOx’s activity remained consistently close to free GOx’s, indicating that the immobilization process had no negative effect on the enzyme’s stability. These encouraging results and the new immobilization approach developed in this study offer various advantages when compared with GOx in its free form and open new horizons for the practical use of GCN-T/GOx in several applications. A promising application of GCN-T/GOx was demonstrated by fabricating a glucose biosensor, which showed a rapid and sensitive response. This work is a breakthrough in the use of GCN-T for the immobilization of enzymes and provides a further opportunity to develop other GCN-T-based bioconjugates.

## Supplementary Material



## Notes

XPS full scan spectra; XPS high-resolution spectra; cyclic voltametric curves at different scan rates; and cyclic voltametric curves at different glucose concentrations (PDF)
