# Supplementary material for: Graphitic Carbon Nitride: A Reusable and Stable Support for Glucose Oxidase
Source: Langmuir. 2025 Jun 17;41(25):15880–91. doi: 10.1021/acs.langmuir.5c00737 (PMC12818753; doi:10.1021/acs.langmuir.5c00737)
Supplement: Supplementary file 1 [file la5c00737_si_001.pdf]

Supporting Information

Graphitic carbon nitride: a reusable and stable support for glucose oxidase

Rita A. M. Barros[1,2], Rivereau Loan[3], Maria J. Sampaio[1,2], Raquel O. Cristóvão[1,2], Cláudia G. Silva[1,2], Joaquim L. Faria[1,2*]

[1]LSRE-LCM – Laboratory of Separation and Reaction Engineering – Laboratory of Catalysis and Materials, Faculty of Engineering, University of Porto, Rua Dr. Roberto Frias, 4200-465 Porto, Portugal

[2]ALiCE – Associate Laboratory in Chemical Engineering, Faculty of Engineering, University of Porto, Rua Dr. Roberto Frias, 4200-465 Porto, Portugal

[3]École polytechnique de l'Université de Lille, Cité Scientifique, Avenue Paul Langevin 59655 Villeneuve d'Ascq Cedex, France

**Corresponding author e-mail: jlfaria@fe.up.pt*

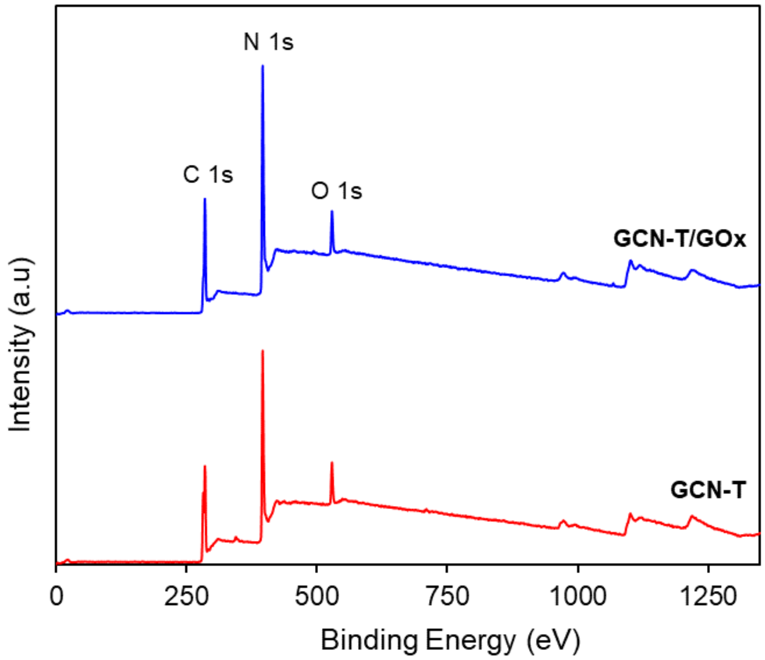

Figure S1. XPS spectra of GCN-T and GCN-T/GOx (full scan).

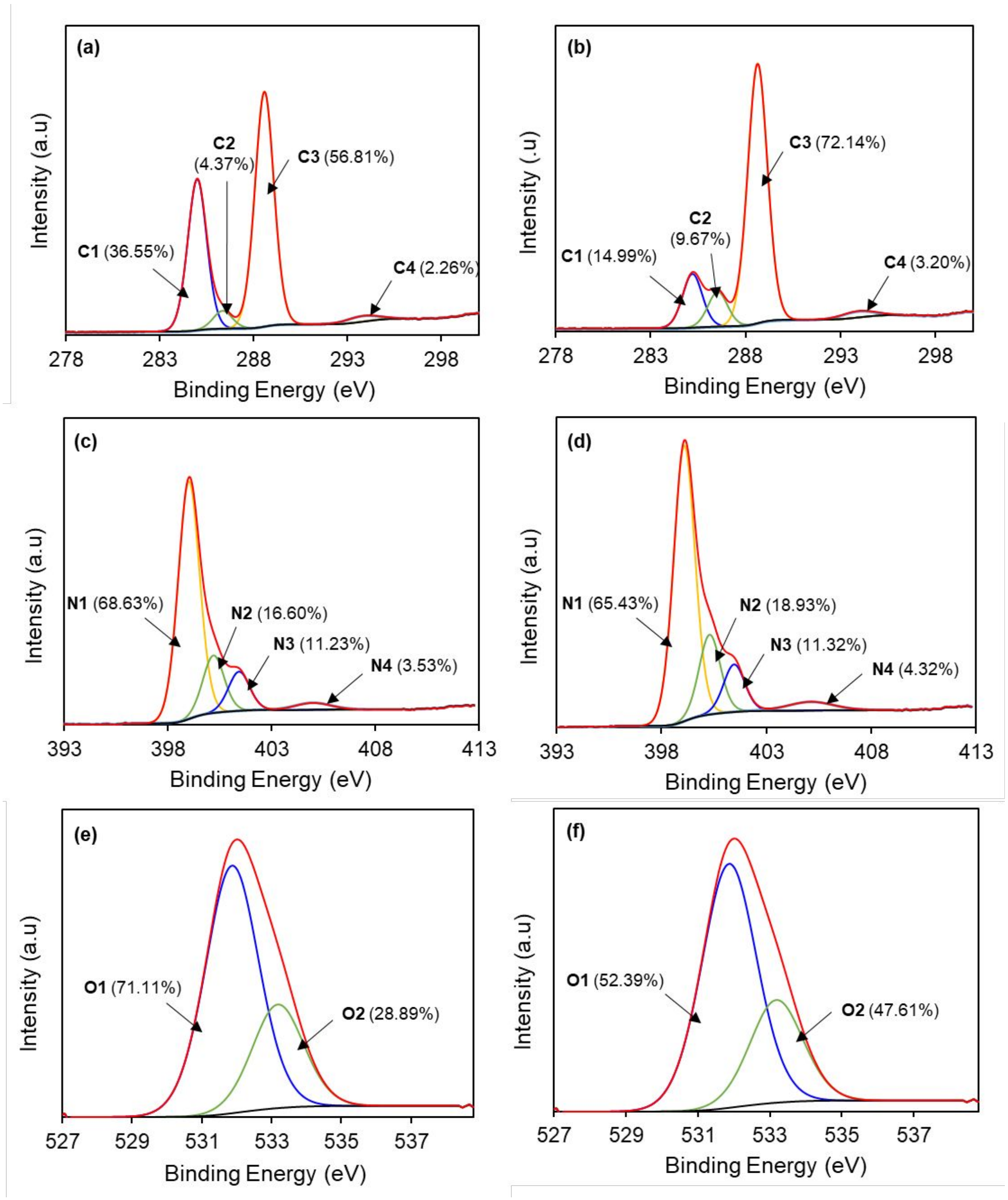

Figure S2. C1s (a, b), N1s (c, d), and O1s (e,f) XPS spectra of GCN-T (a, c, e) and GCN-T/GOx bioconjugate (b, d, f).

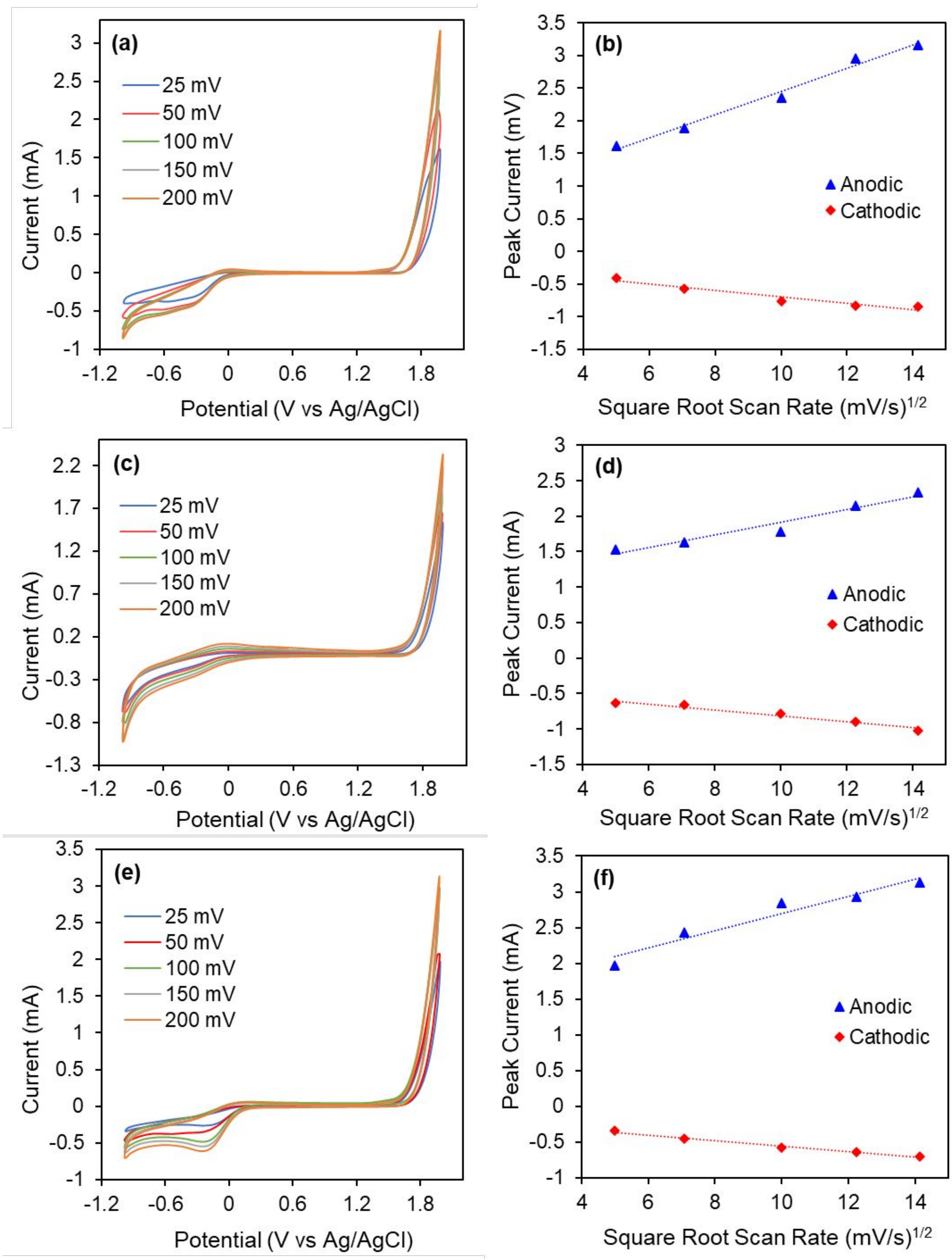

Figure S3. Cyclic voltammetric curves of FTO/Nf/GCN-T (a), FTO/Nf/GCN-T/GOx (c) and FTO/NF/GCN-T/GOx + 5 mM Glucose (e) in 0.15 M PBS (pH 7.4) solution at different scan rates. The corresponding plots of variation between the anodic and cathodic peak current and the square root of the scan rate are shown on the right (b, d, f, respectively).

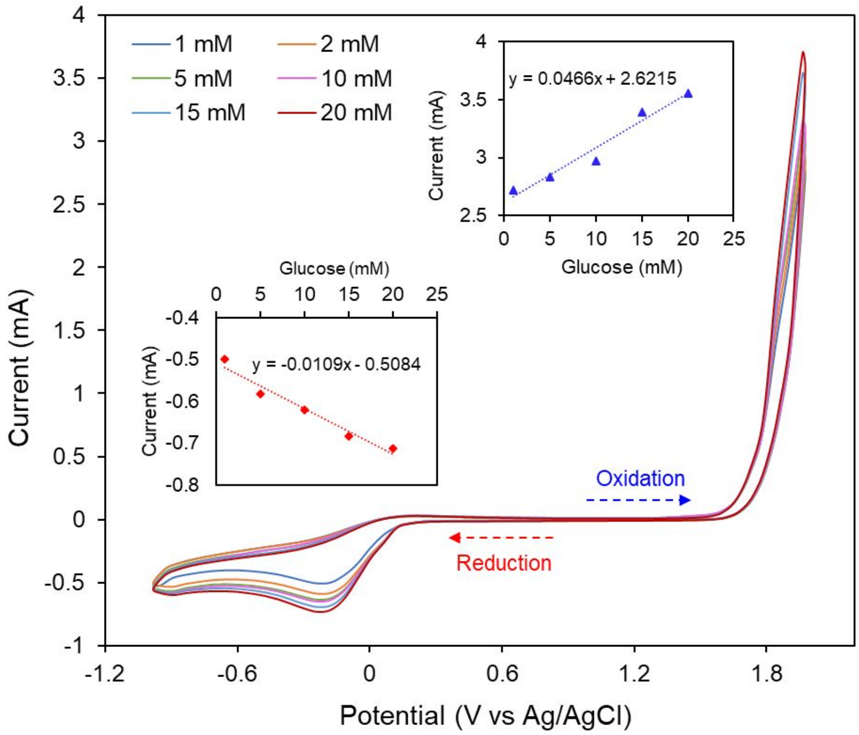

Figure S4. Cyclic voltammetric curves of FTO/Nf/GCN-T/GOx in 0.15 M PBS (pH 7.4) solution in different glucose solutions (at the scan rate of 100 mV s^{-1}).
